# Experiences of being at high-risk during the COVID-19 pandemic and its impact on emotional well-being and daily life in people with chronic conditions: a qualitative study

**DOI:** 10.1186/s41687-023-00607-6

**Published:** 2023-07-05

**Authors:** Caroline Trillingsgaard Mejdahl, Pernille Bjørnholt Nielsen, Lise Arnth Nielsen, Astrid Fyrstenborg Christensen, Berit Kjærside Nielsen

**Affiliations:** DEFACTUM – Public Health Research, Central Denmark Region, Olof Palmes Allé 15, Aarhus N, DK-8200 Denmark

**Keywords:** COVID-19, Risk, Chronic conditions, Everyday life, Emotional well-being, Qualitative, Thematic analysis

## Abstract

**Background:**

With its health risks and extensive disruption to everyday life, the SARS-CoV-2 (COVID-19) pandemic has affected the lives of billions of people. People with chronic conditions are particularly susceptible to severe illness if infected by COVID-19, and they have repeatedly been urged to take stringent steps to ‘shield’ themselves from the virus. It is argued that the negative impact of isolation and other lockdown-related restrictions on emotional well-being and daily life may be most prominent among people at increased risk for severe illness from COVID-19. This qualitative thematic analysis aimed to explore how individuals with chronic conditions perceived the risk posed by COVID-19 and to understand how being at high risk affected their emotional well-being and everyday life.

**Methods:**

The study is a thematic analysis of qualitative data consisting of semi-structured interviews with adults affected by at least one chronic condition supplemented with free text comments from a PRO-based survey.

**Results:**

Based on 17 semi-structured interviews and 144 free text comments from a PRO-based survey three thematic patterns representing diverse COVID-19-related risk experiences were extracted: (1) Feeling vulnerable and at risk, (2) Uncertainty about being at risk, and (3) Distancing from the high-risk label.

**Conclusions:**

The risk of COVID-19 impacted the participants’ everyday lives and emotional well-being in various ways. Some participants felt vulnerable and at risk causing them and their families to take on far-reaching precautions with significant consequences for their everyday life and emotional well-being. Some participants expressed uncertainty associated with whether they were at increased risk. Such uncertainty gave rise to dilemmas about how to navigate their everyday life. Other participants did not identify themselves as at higher risk and took no special precautions. Such a lack of perceived risk may undermine their motivation for taking preventive measures, which calls for public attention regarding current or future pandemics.

## Background

The SARS-CoV-2 (COVID-19) pandemic has affected the lives of billions of people and is considered the most pressing public health challenge of our time [1]. The pandemic is having substantial psychological impacts on populations globally, with increased levels of stress, anxiety, and depression, especially in healthcare workers [2–4] and in people with pre-existing medical conditions [5–7]. In addition to direct health risks from the pandemic, extensive disruption to everyday life occurred worldwide due to widespread containment measures [8]. Such measures included national lockdowns, social distancing, school closures, and quarantines with significant impact on people’s everyday life and emotional well-being [8]. It is argued that such measures are often more burdensome for people most at risk from COVID-19 [8–11]. People with chronic conditions (CC), as well as people with severe obesity and old age, are particularly susceptible to severe illness if infected by COVID-19, and they have an increased risk for hospitalization, intensive care, need for a ventilator and/or death [12]. In Denmark, The Danish Health Authorities likewise identified population groups and individuals most at risk from COVID-19, including people with CCs, people aged 65 + as well as people with severe obesity. In Denmark, almost two-thirds of the entire population aged 16 years or above have one or more chronic condition [13]. Entailing that a very large part of the population in the initial phase of the pandemic was identified as being at potential high risk and on this basis urged to take particularly stringent steps to ‘shield’ themselves from the virus.

Research has established that people with CCs have wide-ranging and profound concerns regarding the impact of COVID-19 [8, 14, 15] and studies have identified high levels of stress and anxiety related to the pandemic, particularly in people with existing medical conditions [6, 7]. Many studies have shown a pronounced fear of transmitting infection and of becoming infected with COVID-19 among people at increased risk of severe illness from COVID-19 [15–18]. In addition, several studies have recognised the negative impact of isolation and other lockdown-related restrictions on emotional wellbeing and daily life among people at increased risk for severe illness from COVID-19 [12, 16, 17, 19, 20]. Thus, several studies have via patient-reported outcome (PRO) measures demonstrated decreases in well-being among people with CC’s during the initial phase of the pandemic and research has shown that people’s risk perceptions of COVID-19 are negatively associated with well-being [21].

Hence, it is important to explore how individuals with chronic conditions perceive the risk posed by COVID-19 and to understand how being at high risk affect their emotional well-being and everyday life.

### Aim

This study aimed to explore perspectives on being at high risk during the initial phase of the COVID-19 pandemic among people living with chronic conditions. Moreover, we aimed to understand how living with the risk of COVID-19 impacted their emotional well-being and everyday life.

## Methods

### Design

The study is designed as a thematic analysis of qualitative data consisting of semi-structured interviews supplemented with free text comments from a PRO-based survey. The thematic analysis is inspired by Braun and Clarke [22].

### Setting

This study is part of the prospective Danish PAM-COVID-19 study aiming to investigate the impact of COVID-19 on well-being among adults with chronic conditions. The PAM-COVID-19 study comprises two sub-studies; (1) A prospective questionnaire survey and (2) a qualitative explorative study. (Results from the PAM-COVID-19 survey will be reported in a separate article).

### Data collection and sampling

Data consisted of free-text comments from the PAM-COVID-19 questionnaire survey and semi-structured interviews.

### Free text comments

The survey data was collected from May 4 to June 1, 2020. In the survey, participants were invited to write additional comments in an unlimited text box. Out of the total 1273 survey participants, 144 supplemented their response with a free text comment. While these free-text comments provided rich and nuanced content, we complemented them with in-depth individual interviews as this type of qualitative data allowed us to delve deeper into the perspectives and aspects expressed in the free-text comments. In addition to being independently included in the thematic analysis, the content of the free-text fields contributed to informing the interview guide.

### Semi-structured interviews

Participants for interviews were identified via the survey in which the participants were asked to indicate if they in addition to the survey, were interested in participating in an interview. Out of 1273, 248 participants consented to participate in an in-depth interview.

Purposive sampling was used to include individuals representing diversity regarding sex, age, type of chronic condition, well-being as measured by the WHO-5 Well-Being Index [23], and perceived level of self-management as measured by the patient activation measure [24]. A data management file was generated based on the above criteria and authors CTM, PBN, or BKN contacted potential participants arbitrarily, all agreed to participate in the interviews.

Because of COVID-19 restrictions, the semi-structured interviews were conducted from a distance by phone from June to August 2020. At that time, there were still COVID-19 restrictions in Denmark; however, de-escalation of containment measures had been initiated. The interviews lasted 27 to 63 min and were all recorded and transcribed verbatim. An interview guide was developed based on an initial analysis of the free text comments and a review of the relevant literature. The themes of the interviews were broad and consisted with the themes in the survey and covered: (1) everyday life, (2) fear and concerns, (3) help and support, (4) physical and mental health issues, and (5) risk perceptions. Using follow-up questions and prompts designed to release participant experiences, participants were invited to share their personal experiences of living with a CC during the first phase of the COVID-19 pandemic. The interviews were conducted by CTM, PBN, and BKN, all with vast experience conducting qualitative interviews.

### Data analysis

The first and last authors performed the data analysis supported by discussions with the research team. We performed a thematic analysis inspired by the analytical steps described by Braun & Clarke [22]; Initially, we read the transcribed interviews several times while noting preliminary ideas to familiarize ourselves with the data. Interesting features across the dataset were then systematically coded, and data relevant to each initial code were carefully collected. Due to the study’s exploratory nature, our initial coding was essentially open. Having arranged our data in meaningful groups, we started identifying themes. At this point, we included the empirical data from the survey sample’s free text comments in our analysis. Next, we reviewed, refined, and defined our proposed themes and subthemes. Finally, we named the themes and subthemes and selected compelling extract examples from interviews and free-text comments that captured the essence of the themes [22]. Data management was facilitated by the qualitative software program NVivo™ [25].

### Ethical considerations

According to Danish law, this kind of qualitative study does not require notification to the Committee on Biomedical Research Ethics. All procedures performed in studies involving human participants were in accordance with the ethical standards of the institutional and national research committee and with the 1964 Helsinki declaration and its later amendments or comparable ethical standards. Informed consent was obtained, and all general requirements for health science research were followed.

## Results

A total of 17 interviews were conducted, representing eight women and nine men aged 39 to 82 years (mean: 59.7). The participants were all affected by at least one CC, such as heart disease, diabetes, chronic lung disease, cancer, or overweight (Table 1). A total of 144 free-text comments were analyzed, and the free-text sample comprised 80 women and 64 men (Table 2).

**Table 1:** Interview participants’ characteristics

			N
**Total (N)**			17
			**n (%)**
**Age, years**			
	< 60		6 (35,3)
	60–69		6 (35,3)
	≥ 70		5 (29,4)
**Sex**			
	Male		9 (53)
	Female		8 (47)
**Primary referral Diagnosis**			
	COPD		39 (19,7)
	Type 2 diabetes		33 (16,7)
	Cardiovascular disease		39 (19,7)
	Cancer		36 (18,2)
	Overweight		16 (8,1)
	Stress or mental illness		12 (6,1)
	Chronic pain		3 (1,5)
	Other		20 (10,1)
**Multi morbidity**			
	No		4
	Yes		10
	*Missing*		*3*
**Well-being**			
	Low risk of depression		5
	Moderate risk of depression		5
	High risk of depression		5
	*Missing*		2
**PAM-level**			
	Level 1 (lowest level of self-management)		5
	Level 2		5
	Level 3		3
	Level 4 (highest level of self-management)		2
	*Missing*		*2*
**Cohabitation status**			
	Living alone		4
	Living with spouse/cohabitant		13
**Educational level**			
	Low (0–10 years of education)		1
	Medium (11–14 years of education)		11
	High (≥ 15 years of education)		3
	*Missing*		*2*
**Employment status**			
	Employed		6 (35,3)
	Unemployed		11 (64,7)

**Table 2:** Free-text participants’ characteristics

			N
**Total (N)**			144
			**n (%)**
**Age**	< 60		56 (38,9)
	60–69		49 (34)
	≥ 70		39 (27,1)
**Sex**			
	Male		64 (44,4)
	Female		80 (55,6)
**Diagnosis**	COPD		24 (16,7)
	Type 2 diabetes		31 (21,5)
	Cardiovascular disease		18 (12,5)
	Cancer		24 (16,7)
	Overweight		18 (12,5)
	Stress or mental illness		13 (9)
	Chronic pain		8 (5,6)
	Other		7 (4,9)
	*Missing*		*1 (0,7)*
**Multi morbidity**			
	No		66 (45,8)
	Yes		74 (51,4)
	*Missing*		*4 (2,8)*
**PAM-level**			
	Level 1 (lowest level of self-management)		33 (22,9)
	Level 2		37 (25,7)
	Level 3		50 (34,7)
	Level 4 (highest level of self-management)		24 (16,7)
**Cohabitation status**			
	Living alone		103 (71,5)
	Living with spouse/cohabitant		36 (25)
	*Missing*		*5 (3,5)*
**Educational level**			
	Low (0–10 years of education)		18 (12,5)
	Medium (11–14 years of education)		73 (50,7)
	High (≥ 15 years of education)		39 (27,1)
	*Missing*		*14 (9,7)*
**Employment status**			
	Employed		57 (39,6)
	Unemployed		82 (56,9)
	*Missing*		*5 (3,5)*

Overall, the thematic analysis displayed that the risk of COVID-19 impacted the participants’ everyday lives and emotional well-being in various ways. In the thematic analysis, we extracted three thematic patterns representing diverse COVID-19-related risk experiences: (1) *Feeling vulnerable and at risk*, (2) *Uncertainty about being at risk*, and (3) *Distancing from the high-risk label* (Table 3). In our analysis, we found no connection between the participants’ WHO5 level, PAM level, number of CC’s, or type of CCs and risk perception. In the following, the three themes and associated subthemes are described in detail and illustrated by participant quotes and free-text comments.

**Table 3:** Findings

Feeling vulnerable and at risk	Uncertainty about being at risk	Distancing from the high-risk label
• Heartfelt fear and social deprivation • Suffering under the precautions of others	• Uncertainty and confusion • Not knowing how to navigate	• High-risk does not apply to me • To worry on behalf of others

### Feeling vulnerable and at risk

#### Heartfelt fear and social deprivation

Almost half of the interview participants felt particularly vulnerable during the pandemic because of an increased risk of a severe course of illness if they got infected with the coronavirus. These participants knew they belonged to a particularly vulnerable high-risk group and took many precautions. One participant said:

*We are still very cautions (…). I miss being with others. Some of my friends have chosen to go out to eat, and I could not join them because I have a chronic lung disease, I have decided to be extra careful. Then I am cut off from those activities because people cannot see that I need to keep a two distance of two meters. I am afraid to sit in a restaurant and be with the others like we have been used to.*

Our analysis displayed that these participants suffered significant social deprivation due to their fear of the coronavirus, which affected their emotional well-being to a great extent. Like in the quote above, our analysis showed that many participants felt insecure in public spaces due to the fear of getting infected, which impacted their everyday lives profoundly. Uncertainty about their CC and its implications in regard to the severity of being infected with the coronavirus meant that the virus was perceived as an invisible enemy. As a consequence, many of these participants refrained from public places when they feared there might be many people. A participant explained:

*It is like having a sack pulled down your head. You go into shock. What happens next? You are powerless, and you cannot do anything. You cannot tell if the person you pass has it (COVID-19). Will you get infected tomorrow? Will you die in a fortnight or three weeks?*

The analysis of the free text comments also revealed how several participants felt vulnerable and exposed due to their CC. One participant wrote:

*I have had type 1 diabetes for over 50 years, and I am 63. I feel very vulnerable when it comes to being in public. It is very insecure.*

Some participants expressed that they believed getting infected with corona would be a certain death. Thus, these participants and their families took extensive precautions in everyday life because they panicked and feared getting infected. A participant alluded to the notion of fear of getting infected:

*I felt scared. It was simply because of my disease. I felt afraid to get it (COVID), right? I know that if I get it, it could mean my death here and now.*

Another participant noted in a free text comment:

*I have multiple diseases. Therefore, and because of my age, I predict that I will die if I get corona. Therefore I take care of myself and find suitable replacements for what I did earlier.*

Daily TV broadcasts from around the world (at that time, notably from Italy) displaying the seriousness of the virus worsened some of the participants’ fear of getting infected and reinforced their experience of being at risk. When asked if he was worried about getting infected, an older man noted:

*Yes, I was pretty afraid of that. I really was. You saw it on television from Italy, where they died like flies. At that time, I was awfully scared to get it (COVID-19) because I knew that then it would be over.*

Accordingly, two interview participants explained how their fear of becoming infected had caused them to refrain from physical contact, even with their spouses, at the beginning of the pandemic. A woman explained:

*At our house, there were no hugs and squeezes and these things. The first month, even my husband and I stopped with hugs and squeezes. We kept that distance (…) because he should not risk having something and bringing it to me.*

For these participants, fear of getting infected had profound impact on their everyday life and their emotional well-being was greatly affected.

#### Suffering under the precautions of others

Finally, our analysis displayed that more participants felt lonely and abandoned because their families refrained from visiting them due to the participant’s potential risk as stated by the health authorities. The families’ kind-hearted considerations resulted in several participants feeling isolated and lonely. An older man said:

*The loneliness was the worst, and it still is (...) My girls do not dare come here because they are afraid of infecting me. I was and still am in the risk group.*

Thus, their emotional well-being seemed to deteriorate further for more participants because the isolation and precautions debarred them from the support and closeness they needed in a time of fear and worry.

### Uncertainty about being at risk

#### Uncertainty and confusion

Across the empirical material, we found various examples of uncertainty associated with whether one belonged to the high-risk group. Especially in the free text comments analysis, we retrieved a rather pronounced uncertainty associated with whether one belongs to a high-risk group or not. Thus, many of the free text comments displayed a general uncertainty as to whether one was at particular risk based on a rather diversified announcement from the public health authorities. One participant noted:

*I am 67 years. In October/November, I got balloon dilations – 4 stents, but I do not feel it, and I am in reasonably good shape and feel super fine, so I am unsure if it means I am in the risk group.*

Due to what the participants perceive as unclear and rapidly changing information from the health authorities, many survey participants seemed doubtful whether they were susceptible to severe illness if infected by COVID-19. A participant commented:

*Regarding chronic conditions, I think that it has been unclear which (for example, sclerosis) are among the special risks.*

#### Not knowing how to navigate

For some participants, this uncertainty about whether or not they belonged to a high-risk group gave rise to dilemmas about how to navigate their everyday life:

*I have diabetes and have been challenged because I lack information from the Danish Health Authority about how people with diabetes should behave. One time you are in a risk group; the next, you are not, but when you read statistics, people with diabetes can become severely ill, so it is pretty insecure not completely knowing whether you should be at home or at work.*

Our analysis further displayed that the interview participants largely recalled such uncertainty about whether one was at risk in the initial phase of the pandemic. A participant said:

*At first, I have to admit that I thought I was one of those who might end up on a ventilator. My doctor could not answer whether that was the case, i.e., whether I was at increased risk. And that uncertainty made me so worried.*

Our analysis displayed that this uncertainty about being at risk affected the participants’ emotional well-being, as they constantly felt that they had to be on guard. Further, the uncertainty caused them to feel insecure about their choices and actions.

### Distancing from the high-risk label

#### Risk does not apply to me

In contrast to the first group’s experiences of being at high risk, our analysis also displayed a widespread distancing from belonging to a high-risk group for some participants. Although all participants, by definition, belonged to a high-risk group due to their chronic condition and or high age, more than half of the interview participants did not identify themselves as someone at higher risk. A participant uttered:

*I had a blood clot about half a year ago, so if it should be correct, then I probably belong to some risk group, but now I have chosen to ignore that because I am not sick and will not become it.*

As seen in the above quote, more participants perceived it as being up to one’s own assessment of whether one belongs to a high-risk group. If the participants generally felt well and in good health, we found a distinct tendency for them to distance themselves from the risk labeling. This tendency is also retrieved in the analysis of the free text comments. A survey participant noted:

*I am overweight, and my BMI is 38,7. But aside from this, I am healthy (…). I have read that overweight people have a greater risk of the corona, but I do not consider myself a lousy risk group and believe I can do much myself with good hand hygiene.*

Thus, several participants argued against having an increased risk of serious illness if they got infected based on an experience of not feeling particularly ill in general. Not surprisingly, our analysis showed that this distancing from risk meant that their emotional well-being and everyday life were not as affected as those who felt at significant risk. To the question of whether she felt at particular risk of COVID-19 due to her being overweight, a woman stated:

*Actually, not really, because I do not feel sick. I do not see my overweight as a disease. I see it as an annoying thing I have struggled with for many years. So I am actually fresh and healthy. So no, I have not thought that. In many aspects, I am hundreds of times healthier than other people I know. It is just some annoying weight I cannot get rid of (…). I actually feel that my health is good enough. But I have been more afraid of dragging it (COVID-19) with me and infecting others.*

As seen in the above quote, another typical explanation for why participants did not feel particularly at risk was that they often compared themselves to someone in their circle of acquaintances whom they believed was worse off.

#### To worry on behalf of others

The analysis further displayed that more participants, like the woman in the quote above, express far more significant concerns for the well-being of others than for their own health, despite suffering from one or more chronic conditions. To the question of what had been her biggest concern during the pandemic, a participant explained:

*Well, my family should get sick, especially those particularly vulnerable. I know that at my age, I should also be a little vulnerable, but I think that I worry more about my daughter-in-law and son-in-law and daughter, who I think is a little more exposed and vulnerable.*

Thus, we found that several participants seemed more focused on the risk profile of their loved ones than their own risk profile. A participant noted in a free text comment:

*My biggest worry is that my son has a hereditary heart condition, and I will do everything to avoid that he gets infected.*

In continuation of this concern of others, the analysis showed that several participants expressed significant concerns about the risk of being a carrier of the virus without knowing and thus potentially risking infecting vulnerable family members and other acquaintances.

When asked if she were afraid of getting infected with corona, a woman uttered:

*Well, I did not think much about being afraid of it. I was more fearful of infecting others. I mean, we could be healthy carriers of the infection. That is what has been the worst. That was what I thought if you could become a healthy carrier of the infection.*

Our analysis pointed out that a common feature among the participants who do not perceive themselves as being at high risk is a general experience of not being so bothered by their chronic illness condition. Although this group of participants did not feel particularly at risk, for a large part of them, their emotional well-being was still affected because they worried about their loved ones’ risk of getting infected.

## Discussions

The present study comprehensively explored the everyday life experiences of adults living with CCs in response to the early phases of the pandemic and its impact on their emotional well-being. The analysis displayed somewhat diverging perspectives on belonging to a high-risk group. Some participants felt very vulnerable and at risk due to their CC. In the analysis, we point to a genuine fear of contracting the virus among these participants, causing them and their families to take on far-reaching precautions to avoid infection. This finding broadly supports previous research into COVID-19-related concerns in people living with CCs during the pandemic [8, 15, 18]. A scoping review on the social and psychological impact of the COVID-19 pandemic on people with Parkinson’s disease found that participants reported various concerns about the pandemic, particularly fear of infection, as well as fear of requiring intensive hospital care if they were to become infected [18]. An interview study documented that living with the threat of being infected with coronavirus has negatively affected everyday life for patients with chronic obstructive lung disease [17]. Another qualitative study on the impact of the pandemic on the mental health and well-being of people living with CCs found high levels of fear and anxiety related to the perceived consequences of COVID-19 infection [15]. In line with our findings, Fisher et al. further found that some participants who were shielded suffered particularly from social isolation [15]. Our study showed that participants who felt at high risk significantly reduced the number of their social contacts. This finding is echoed greatly by risk perception research results concerning the COVID-19 pandemic. Thus, several studies have established the association between high-risk perception towards COVID-19 and keeping a social distance [26–28]. In a longitudinal survey study on the influence of risk perceptions on close contact frequency during the pandemic it was documented that people who perceived themselves to experience severe illness if thy contract a COVID-19 infection tended to make significantly fewer contacts as compared to those who had low or neutral perceptions [27].

Contrary to expectations, our study also found that several participants did not identify themselves as being at higher risk. Accordingly, these participants did not express fear and anxiety related to the perceived consequences of COVID-19 infection. Their emotional well-being and everyday life were less affected than the group of participants who largely considered themselves at risk. This “lack” of perceived risk and absence of COVID-19-related concern can be interpreted as optimism bias, a phenomenon where people tend to underestimate their likelihood of experiencing an adverse event or overestimating the likelihood of positive events [27, 29]. Such underestimation of perceived risk in the context of the COVID-19 pandemic has been retrieved in other studies [27, 30, 31] emphasising the complex nature of risk perception. In the PAM COVID-19 survey, similar results were found. More than a fifth did not perceive themselves to be at particular risk of becoming seriously ill if they were infected with the coronavirus [14]. These findings raise an interesting question about the classification of citizens by the healthcare system and authorities with whom they do not necessarily identify, even during a worldwide and potentially life-threatening pandemic. The divergence between the healthcare system’s perception and the citizens’ perception is significant, mainly because this can lead to a higher willingness to take risks among those who either have doubts or do not believe they are at particular risk [14, 32]. Thus, our study adds to the understanding of the diverse impact of the COVID-19 pandemic on people living with chronic conditions.

### Recommendations

Based on the findings, the following recommendations can be made for public health agencies:

*Provide targeted support and guidance*: Recognize that individuals who perceive themselves as high-risk require specific support. Public health agencies should develop tailored guidance and resources to address the concerns and uncertainties faced by this group. This may include clear communication about risk factors, preventive measures, and available support services.

*Enhance risk communication*: Public health agencies should strive to improve communication efforts to ensure accurate and accessible information reaches all segments of the population. Emphasize the potential risks associated with COVID-19, particularly for individuals who may not perceive themselves as high-risk. This can help motivate individuals to take necessary preventive measures.

*Promote psychosocial support*: Recognize the emotional impact of the pandemic on individuals at high-risk. Public health agencies should collaborate with mental health professionals to offer counselling services, support groups, and other resources to address the emotional well-being of these individuals and their families. This support can help alleviate anxiety and provide coping strategies during challenging times.

There are some limitations to our study. Qualitative telephone interviews are often depicted as the less attractive alternative to face-to-face interviews due to the absence of visual cues via telephone, resulting in a loss of contextual and nonverbal data [33]. However, the empirical material revealed broad and nuanced participant perspectives on how participants had experienced living with a CC during the initial phase of the pandemic. Further, interview data were only collected at a single interview relatively early during the pandemic. Having performed follow-up interviews later during the pandemic would add another layer to understanding how the risk of COVID-19 impacted the participants’ everyday lives and emotional well-being. However, the time shift between our two data collections (free-text collected in May 2020 and interview data collected June-August 2020) also constitutes a strength in the study’s design, as it contributes a nuanced picture of how being a risk was experienced. Finally, we found no connection between the participants’ well-being, PAM level, and risk perception. This may be due to the relatively small sample of 17 participants. If more participants had been included, a correlation between PAM, well-being, and risk perception might have been found.

## Conclusions

This study draws attention to how individuals with chronic conditions perceive the risk posed by COVID-19. The findings demonstrate that the risk of COVID-19 impacted the participants’ everyday lives and emotional well-being in various ways. Some participants felt vulnerable and at risk causing them and their families to take on far-reaching precautions with significant consequences for their everyday life and emotional well-being. Some participants expressed uncertainty associated with whether they were at increased risk. Such uncertainty gave rise to dilemmas about how to navigate their everyday life. Other participants did not identify themselves as at higher risk and took no special precautions. Such a lack of perceived risk may undermine their motivation for taking preventive measures, which calls for public attention regarding current or future pandemics.

## Data Availability

The datasets generated and analysed during the current study are not publicly available due to the confidential nature of the qualitative data, but are available from the corresponding author on reasonable request.

## Abbreviations

CC: chronic conditions
COVID-19: SARS-CoV-2
PRO: Patient-Reported Outcomes
